# A Novel General (*n*, *n*)-Threshold Multiple Secret Images Sharing Scheme Based on Information Hiding in the Sharing Domain

**DOI:** 10.3390/e24030318

**Published:** 2022-02-23

**Authors:** Fengyue Xing, Xuehu Yan, Long Yu, Longlong Li

**Affiliations:** 1 College of Electronic Engineering, National University of Defense Technology, Hefei 230037, China; xingfy@nudt.edu.cn (F.X.); yl@nudt.edu.cn (L.Y.); lilongs@nudt.edu.cn (L.L.); 2 Anhui Province Key Laboratory of Cyberspace Security Situation Awareness and Evaluation, Hefei 230037, China

**Keywords:** secret image sharing, multiple secret images sharing, random element utilization model

## Abstract

(k,n)-threshold secret image sharing (SIS) protects an image by dividing it into *n* shadow images. The secret image will be recovered as we gather *k* or more shadow images. In complex networks, the security, robustness and efficiency of protecting images draws more and more attention. Thus, we realize multiple secret images sharing (MSIS) by information hiding in the sharing domain (IHSD) and propose a novel and general (n,n)-threshold IHSD-MSIS scheme (IHSD-MSISS), which can share and recover two secret images simultaneously. The proposed scheme spends less cost on managing and identifying shadow images, and improves the ability to prevent malicious tampering. Moreover, it is a novel approach to transmit important images with strong associations. The superiority of (n,n)-threshold IHSD-MSISS is in fusing the sharing phases of two secret images by controlling randomness of SIS. We present a general construction model and algorithms of the proposed scheme. Sufficient theoretical analyses, experiments and comparisons show the effectiveness of the proposed scheme.

## 1. Introduction

Secret image sharing (SIS) is a significant branch in multi-party security cryptosystems [1,2], which originates from secret sharing (SS) introduced by Shamir in 1979. (k,n)-threshold SS can process private data into *n* shares managed by different members. When *k* or more members provide their shares, the private data can be recovered. Otherwise, nothing will be revealed about the data. As a special format of data, images seldom raise suspicion by the attackers and can cover much information. Thus, SIS realizes information sharing based on images. In 2002, Thien and Lin realized SIS for the first time [3]. Many researches focus on SIS with various realizations [4,5,6], and continuously study the applications [7,8,9] and improvements [10,11,12,13]. With the development of network technology, users are increasingly demanding the security, robustness and efficiency of secret image transmission. When plenty of secret images are delivered by SIS in cloud computing, it is difficult for users to manage and search among shadow images which are all noise-like and indistinguishable. These shadow images also suffer from the risk of being tampered with. Suppose attackers modify shadow images, secret images cannot be reconstructed correctly. However, the transmission of fake shadow images wastes lots of time and money, which results in a weak efficiency. For unpublished or confidential drawings like industrial products and instructions, it is necessary to ensure the security. There is an intensely close connection among them, but separate encryption and transmission for each drawing will raise the risk of being attacked. In addition, in police, procuratorate, court and other judicial departments, the most common evidence format is the photo or image. As the quantity of evidence is overgrowing, the staff must attach importance to their management, retrieval, storage and security. However, conventional methods like encryption and marking are incapable of balancing the effectiveness and safety and preventing malicious tampering.

**Figure 1:**
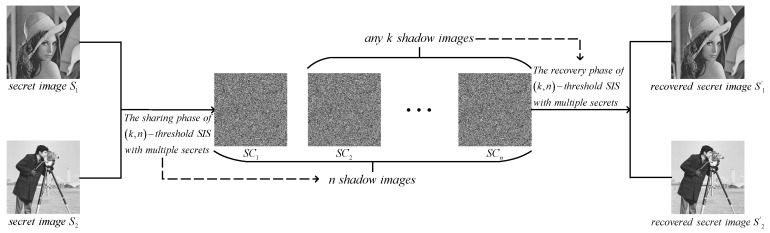
The diagram of MSIS.

The above issues can be addressed by SIS for multiple secrets (MSIS). Existent SISs mainly include polynomial-based SIS (PSIS), Chinese remainder theorem-based SIS (CRTSIS) and visual cryptography (VC). In general, the object of a traditional SIS is one secret image, while MSIS can share two secret images simultaneously. As shown in Figure 1, both secret images are shared at the same time. We obtain *n* shadow images. Any *k* or more of them can recover two secret images. Compared with SIS, MSIS further reduces the risk, improves the effectiveness and saves the storage.

There are broad applications of MSIS. In 2006, Iwamoto et al. [14] constructed a VC scheme (VCS) for multiple secret images in which each shadow image can be rotated with 180 degrees in decryption. However, the reconstructed secret images were with loss. Naidu et al. [15] designed a secure e-voting system which provided authentication based on non-transferable personal credentials like biometric features by MSIS, but its theory presented in [16] also led to a serious loss for recovered fingerprint images and photos. Chen et al. [17] proposed a new boolean-based MSIS scheme (MSISS) to share different sized secret images, but its process was too complicated and the robustness was poor. Sridhar et al. [18] proposed an enhanced (k,n)-threshold MSISS which uses circular shadow images instead of rectangular ones based on random grids; however, it took four stages to complete the scheme with high operational complexity. Prasetyo et al. [19] focused on color secret images and introduced a (n,n)-threshold MSISS. The security of the scheme mainly relied on the randomness of generalized chaotic image scrambling. However, the scheme itself would not solid and safe. In 2020, Chen et al. [20] studied boolean-operation for MSIS and introduced a general access structure, but the general structures of other MSIS principles do not be proposed yet. Wang et al. [21] proposed a polynomial-based scheme to share multiple secret images both within a group and between groups for access control, but the applications of the scheme was too limited. Liu et al. [22] introduced a $\left(k_{1},k_{2},n\right)$-threshold two-in-one secret image sharing scheme with PSIS and random grid-based visual cryptography scheme (RGVCS) for multiple secrets, but the payload was not ideal. In the following sections, we will compare the scheme with our proposed scheme in detailed parameters, which can prove the improved performance of our scheme. There are many issues in the above MSISSs, namely, recovered secret images distortion, the weak security and the poor ability to resist attack, high complexity (complicated steps or phases of a scheme) and the lack of general structures for other principles, and so we proposed a general and novel MSISS to improve the above issues.

In addition, in 2014, Chang et al. [23] proposed a lossless secret sharing scheme using a steganography technique. The scheme combined SIS with steganography. From the idea, Mao et al. [24] proposed a lossless image morphing algorithm to recover the original image from the morphed image. Indeed, the morphed image carried information of two images. However, the scheme realized by combining two independent processes, first realizing SIS and then adding steganography to SIS, which resulted in a low efficiency. For improvement, our proposed scheme is an undivided process by fusing SIS with steganography.

Thus, based on information hiding in sharing domain (IHSD), a (n,n)-threshold MSISS (IHSD-MSISS) is proposed in this paper, and we provide a general construction model and algorithms. The proposed (n,n)-threshold IHSD-MSISS is a general and applicable scheme with users’ inputs, which is an undivided process with a reasonable efficiency. The proposed scheme can improve the existent MSISSs, such as the recovery for original images without loss and low complexity, and it also strengthens the security and the robustness.

In practical applications, for images with a strong correlation conventional methods cannot guarantee the required security. Each separate protection and communication for images will occupy the resource and face unpredictable risk. For instance, once an undisclosed product and its instruction manual are stolen, the company will suffer huge financial and reputation losses. For another example, the police usually takes evidence for photos in the crime scene. If one of several related photos is revealed or modified, the pressure and influence will be tremendous.

The proposed scheme is precisely applicable to the above situations. The company can adopt the proposed scheme, embedding product drawings into instruction drawings. It is more safe and reliable for the company to utilize *n* shadow images and protect these drawings, and they can be recovered at the same time. For police, the proposed scheme can promote the protection for strong-associated evidence and prevent malicious tampering or revelation. The evidence will be divided as *n* shadow images and can be recovered simultaneously. From the above, we can find that the proposed scheme also saves the storage. A traditional (k,n)-threshold SIS of two secret images requires 2×n shadow images for storage, while our proposed scheme occupies half of the space to share two secret images.

Moreover, we can utilize the proposed scheme to manage and identify shadow images. By embedding logo images into shadow images, the efficiency for management and identification will be improved. The proposed scheme is also applicable in covert communication where the more critical secret image can be transmitted in private by a common image.

In this paper, we propose a general (n,n)-threshold IHSD-MSISS to improve existent MSISSs, which based on IHSD [25] is a novel and undivided MSISS. We introduce the general construction model and algorithms of the proposed scheme. Then, with concrete examples, we present sufficient theoretical analyses, experiments and comparisons which prove the effectiveness of the proposed scheme.

The rest of this paper is organized as follows. Relevant basic knowledge for the proposed scheme is presented in Section 2. Section 3 introduces the general and novel (n,n)-threshold IHSD-MSISS and provides the realization steps and algorithms of the proposed scheme. With concrete examples, theoretical analyses and proof are given in Section 4. Section 5 shows the experimental results and comparisons. Finally, Section 6 concludes our work.

## 2. Preliminaries

This section studies existent (k,n)-threshold SISs in detail and concludes their typical characteristics. Then, we review the knowledge of IHSD, which is the basis for our work.

### 2.1. Random Elements in (k,n)-Threshold SIS

Existent popular (k,n)-threshold SISs mainly include PSIS, CRTSIS and VC. (k,n)-threshold SIS follows the principle of dividing a secret image into *n* shares. The secret image can be reconstructed as *k* or more shares are gathered. However, fewer than *k* shares cannot obtain anything about the secret image.

Shamir introduced SS and proposed a scheme based on polynomial interpolation [26], which has been applied in images as PSIS. With simple operation, PSIS is realized by a random (k−1)-degree polynomial from Shamir’s idea, shown as Equation (1). There are prime *p* and *k* coefficients denoted by $a_{0}$, $a_{1}$, ⋯, $a_{k-1}$. $a_{0}$ depicts one pixel of the secret image. The other coefficients are chosen randomly in [0,p) so that the quantity of possible polynomials for one secret pixel is $p^{k-1}$. As *n* different ID numbers $x_{i}\left(1\leq i\leq n\right)$ are inputted in the polynomial, we can obtain *n* final shadow pixels. For secret pixels with the same value, the values of coefficients except $a_{0}$ in the polynomial will be different every time. Thus, we cannot deduce the real secret pixel from each result of shadow images. The above calculations are in the finite field of GF(p). According to the value of *p*, actual schemes can be achieved without loss.

(1) $$f\left(x\right)=\left(a_{0}+a_{1}x+\cdots +a_{k-1}x^{k-1}\right)\;\mathrm{mod}\;p$$

(k,n)-threshold CRTSIS utilizes Chinese remainder theorem and divides pixels of the secret image into two intervals according to two available mapping intervals [6], $\left[T+1,\left\lfloor \frac{M}{p}-1\right\rfloor \right]$ and $\left[\left\lceil \frac{N}{p}\right\rceil ,T\right)$ respectively, which are subject to limitations shown in Equation (2). Corresponding to the range of each secret pixel denoted by *x*, we can decide *A*, which is randomly chosen from the respective mapping interval. This procedure provides plenty of available sharing values for shadow pixels. Then we calculate shadow pixel SC≡y(mod $m_{i})$ and finally obtain *n* shadow images. Relevant parameters are computed in Equation (3). Here, *p* and *T* are public for all members.

(2) $$\left\{\begin{array}{c} \{p,m_{i}|128\leq p<m_{1}<m_{2}<\cdots <m_{n}\leq 255,1\leq i\leq n\},\\ \gcd\left(m_{i},m_{j}\right)=1\left(i\neq j\right),\;\gcd\left(m_{i},p\right)=1\left(1\leq i\leq n\right),\\ M=\prod_{i=1}^{k}m_{i},\;N=\prod_{i=1}^{k-1}m_{n-i+1},\;M>pN,\\ T=[\frac{\left\lfloor \frac{M}{p}-1\right\rfloor -\left\lceil \frac{N}{p}\right\rceil }{2}+\left\lceil \frac{N}{p}\right\rceil ] \end{array}\right.$$

(3) $$\left\{\begin{array}{c} A\in [T+1,\left\lfloor \frac{M}{p}-1\right\rfloor ]\;\mathrm{and}\;y=x+Ap,\;0\leq x<p\\ A\in [\left\lceil \frac{N}{p}\right\rceil ,T)\;\mathrm{and}\;y=x-p+Ap,\;p\leq x\leq 255 \end{array}\right.$$

Among existent popular (k,n)-threshold SISs, PSIS and CRTSIS rely on strong computing ability, while VC can be realized with no computation device. (k,n)-threshold VCS depends on human visual system by stacking or XOR operation for recovery, and it mainly contains basic matrix-based VCS [27,28] and RGVCS [29,30]. Both principles based on a basic matrix and random grid provide multiple alternatives to encrypt the secret pixel and guarantee that the final recovery result by stacking or XOR operation can be distinguishable visually. From the above, we can conclude that SIS provides many available random elements in the sharing phase, which achieves the same effect as confusion.

### 2.2. IHSD

**Definition** **1** (Information hiding in the sharing domain [25])**.** *Information hiding in the sharing domain utilizes the sharing phase of SIS to share a secret image and hide extra information at the same time. The recovered information and the secret image will be obtained simultaneously, but the extraction of information and the recovery of the secret image are separate operations. In addition, IHSD follows the conditions:*

*1*. ***Security condition.** Only by using k or more shadow images can a secret image be recovered. Otherwise, they do not contain any of the content of the secret image.*
*2*. ***Secret recovery condition.** Among n shadow images, at least k of them can be used to recover the secret image.*
*3*. ***Information hiding condition.** IH in IHSD is reversible. The embedding and extraction of hidden information are realized in the sharing and recovery phases of the secret image, respectively.*

IHSD is a novel definition that we utilize random elements generated from the sharing phase of SIS and consider them the sharing domain. Any secret image can be shared and any form of extra information can be communicated silently at the same time in this domain. As a general and inclusive method, IHSD studies the availability of random pixels during the sharing process of SIS to protect the secret image and simultaneously hide extra information by screening. IHSD first introduces the sharing domain, where we originally fuse independent procedures of SIS and information hiding into one whole phase instead of a simple combination. Owing to the inclusiveness of IHSD, it is applicable for practical applications such as law enforcement and medical diagnoses and results in a better performance on security and efficiency.

SIS is a reversible process, and the sharing phase is symmetrical to the recovery phase. Thus, we apply SIS as the third condition to IHSD and propose a novel and general MSISS.

## 3. The Proposed (n,n)-Threshold IHSD-MSISS

In this section, the proposed (n,n)-threshold IHSD-MSISS is introduced, presenting the design concept and theoretical realization, and we introduce the corresponding algorithms with detailed comments. Then, we provide the evaluating metrics which can measure the proposed scheme objectively and effectively. An example is discussed for better understand. Finally, we specifically stress the highlights of the proposed scheme. Strengths and weaknesses are also given.

### 3.1. Introduction of (n,n)-Threshold IHSD-MSISS

(n,n)-threshold IHSD-MSISS is a novel and general MSISS, where we can input an SISS and two legal secret images. The proposed scheme can share two secret images into *n* shadow images. In the recovery phase, both secret images will be obtained simultaneously.

#### 3.1.1. Design Concept and Realization

(n,n)-threshold IHSD-MSISS is mainly based on IHSD. By utilizing randomness of SIS, IHSD controls the available shadow pixels to share a secret image and at the same time hides extra information. (n,n)-threshold IHSD-MSISS makes shadow images as extra information of IHSD to share two secret images. In addition, an SISS can process a secret pixel into *n* shadow pixels, while (n,n)-threshold IHSD-MSISS focuses several pixels at the same time (assuming that there are *J* pixels) and operates on bits.

(n,n)-threshold IHSD-MSISS realizes in the following steps:

1. Between two secret images, one will be considered the primary secret image, while the other will be the secondary secret image.
2. (n,n)-threshold IHSD-MSISS aims at *J* pixels of primary secret image. By an SISS, *J* pixels are shared into *J* different sets of *n* shadow pixels.
3. Among J×n shadow pixels, the proposed scheme selects *m* front bits of each shadow pixel, and combine them in the designed order, which is presented as Algorithm 1. Here, these *m* bits are called *m*-bit payload. The relationship of *J* and *m* is ⌊J×m⌋=8. Then, there are new combinations $C_{1}$, $C_{2}$, ⋯, $C_{n}$ with 8 bits.
4. Try *n* new combinations together to recover a pixel of the secondary secret image. If they can reconstruct the pixel, the proposed scheme will focus on subsequent *J* pixels of the primary secret image and repeat the above steps. Otherwise, the current *J* primary secret pixels will be shared by the SISS again. Bits of shadow pixels generated from *J* primary secret pixels will be recombined. The new combinations will try to recover the pixel of the secondary secret image.
5. After the last pixel of primary secret image is processed, the sharing phase of two secret images finishes. Finally, we obtain *n* shadow images which are with the same type and size as the primary secret image.
6. In the recovery phase, when *n* shadow images are gathered, two secret images will be reconstructed simultaneously.

**Algorithm 1:** The generation of new combinations.
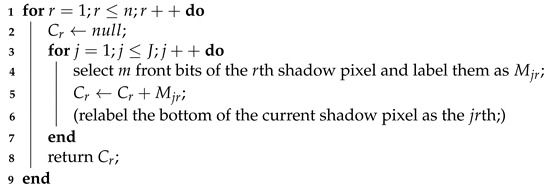


Regarding the steps of the realization, there are several comments as follows:

- In (n,n)-threshold IHSD-MSISS, steps from 1 to 5 are the sharing phase, which generates shadow images which involves the content of two secret images. Step 6 is the recovery phase. Two secret images can be reconstructed simultaneously, and each of them can be recovered individually. When applying (n,n)-threshold IHSD-MSISS, it is for the user to choose an SISS and m-bit payload, provide two secret images and determine which one is the primary secret.
- By an SISS, (n,n)-threshold IHSD-MSISS controls the randomness of the sharing phase of the primary secret, and it combines out the shadow images of the secondary secret to complete its sharing. From another viewpoint, the proposed scheme fuses the sharing phase of the secondary secret image into the sharing phase of the primary secret image, which is an undivided process for sharing multiple secrets simultaneously.
- The main process of (n,n)-threshold IHSD-MSISS is the sharing phase of the primary secret by an SISS. Thus, in the end of the sharing phase, we will obtain the shadow images which are with the same type and size as the primary secret image, which explicates the step 5. For instance, if the primary secret image is a binary image with W×H, *n* shadow images will be binary with a size of W×H.
- In step 3, the proposed scheme choose *m* front bits of shadow pixels to combine, rather than last or random positions. When shadow images are transmitted, some lowest bits of them are so frail that they are easily lost or attacked. If the proposed scheme considers the lowest bits as payload position (or called embeddable position) for combination, the risk of failing to recover secret images will increase. In addition, (n,n)-threshold IHSD-MSISS is reversible. If the proposed scheme takes bits of random positions for combination, the secondary secret image cannot be recovered. Thus, the proposed scheme focuses on the front positions where the bits are robust.
- In step 3, we simply indicate the relationship between *m* and *J*. Image pixels are computed as 8 bits in binary. As *m* bits of front position is selected to embed, $\left\lceil \frac{8}{m}\right\rceil$ pixels of the primary secret image will be processed at the same time (⌊J×m⌋=8, so that $J=\lceil \frac{8}{m}\rceil$). In the sharing phase, the proposed scheme generates *n* shadow pixels from $\left\lceil \frac{8}{m}\right\rceil$ primary secret pixels, then selects *m* front bits for combination. Thus, there are $\lceil \frac{8}{m}\rceil \times m$ payload bits for one pixel of secondary secret image. If the quantity is larger than 8, we merely remain the front 8 bits, which will be combined as a new pixel value in binary. Thus, in final, $\left\lfloor \left\lceil \frac{8}{m}\right\rceil \times m\right\rfloor$ front bits of shadow pixels of the primary secret image are the payload for the secondary secret.
- Steps 3 and 4 are the most time-consuming among all the steps. By the randomness of SIS, we realizes the proposed scheme. From the combination process in step 3, we can know that the number of replication is $n\times J=n\times \lceil \frac{8}{m}\rceil$, so the complexity is $O\left(n\times \left\lceil \frac{8}{m}\right\rceil \right)=O\left(\frac{n}{m}\right)$. With the judgment in step 4, step 3 probably repeats many times. Thus, the complexity of the proposed scheme is $O(\frac{n}{m})$.

The above presents the design concept and general steps of (n,n)-threshold IHSD-MSISS. More detailed realization is presented in the following algorithms.

#### 3.1.2. Algorithms

**Figure 2:**
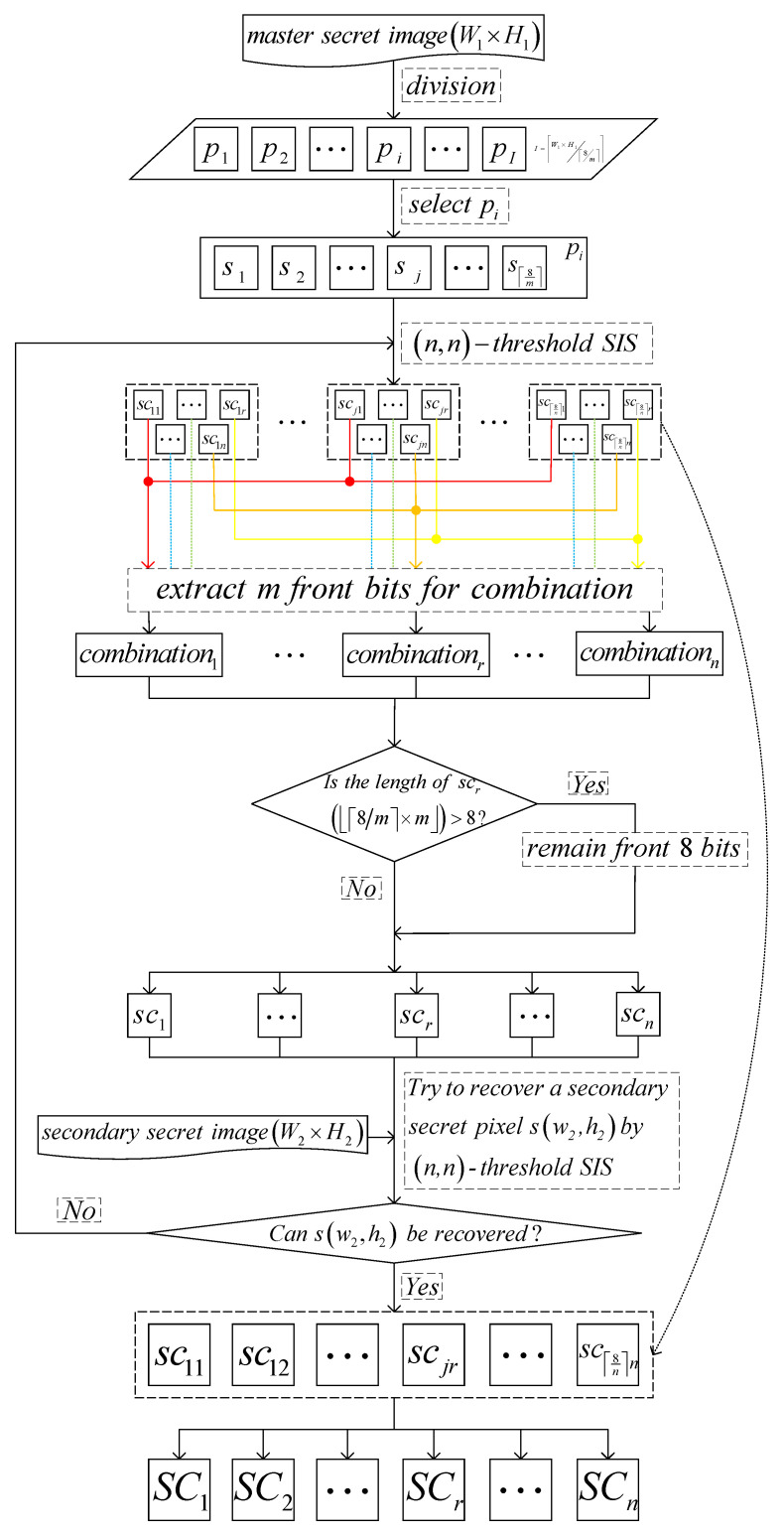
The proposed (n,n)-threshold IHSD-MSISS.

**Figure 3:**
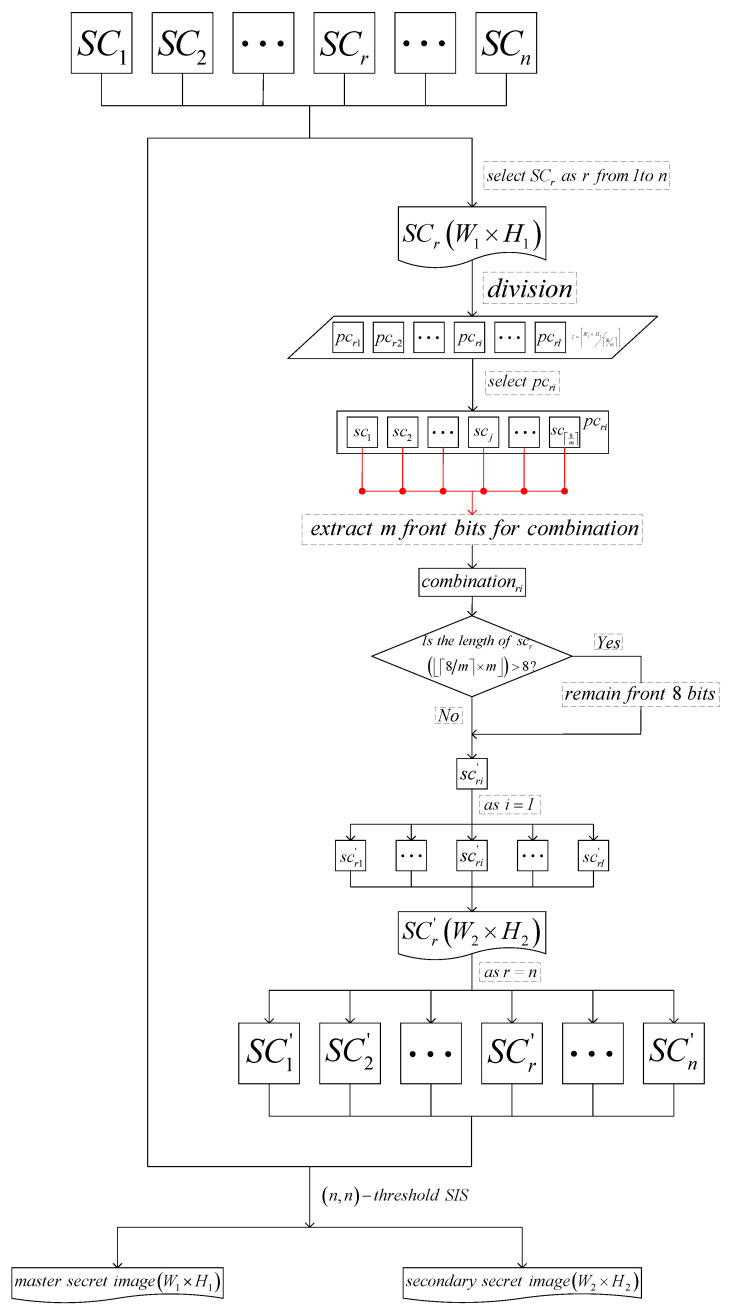
The recovery of the proposed (n,n)-threshold IHSD-MSISS.

(n,n)-threshold IHSD-MSISS includes sharing and recovery phases which are separately presented in Algorithms 2 and 3, and we provide the corresponding flow charts shown in Figure 2 and Figure 3.


**Algorithm 2:** The Proposed (n,n)-threshold IHSD-MSISS.
**1** **Input:** an SISS; a legal secret image $S_{1}$ with a size of $W_{1}\times H_{1}$ (as the primary secret image); a legal secret image $S_{2}$ with a size of $W_{2}\times H_{2}$ (as the secondary secret image); the number of payload bits *m*.**2** **Output:***n* shadow images $SC_{r}\left(1\leq r\leq n\right)$ with a size of $W_{1}\times H_{1}$.**3** **Step 1:** In $S_{1}$, each time fetch continuous $\left\lceil \frac{8}{m}\right\rceil$ secret pixels until the last one, which are as the *i*th segment denoted by $p_{i}$, and there are $\left\lceil \frac{W_{1}\times H_{1}}{\left\lceil \frac{8}{m}\right\rceil }\right\rceil$ segments in total.**4** **Step 2:** For each segment $p_{i}\in \left\{p_{i}|1\leq i\leq \left\lceil \frac{W_{1}\times H_{1}}{\left\lceil \frac{8}{m}\right\rceil }\right\rceil \right\}$, repeat Steps 3–5.**5** **Step 3:** For each pixel $s_{j}\in \left\{s_{j}|1\leq j\leq \left\lceil \frac{8}{m}\right\rceil \right\}$ in $p_{i}$, use the designed random elements utilization model to generate $\lceil \frac{8}{m}\rceil \times n$ shadow pixels $sc_{jr}\in \left\{sc_{jr}|1\leq j\leq \left\lceil \frac{8}{m}\right\rceil ,1\leq r\leq n\right\}$.**6** **Step 4:** Focus on *j* from 1 to $\left\lceil \frac{8}{m}\right\rceil$, extract front *m* bits from each $sc_{jr}$ and combine them sequentially to become a new 8-bit value in binary. If $\lfloor \left\lceil \frac{8}{m}\right\rceil \times m\rfloor >8$, remain the front 8 bits. There are *n* new values in total, denoted by $sc_{r}\in \left\{sc_{r}|1\leq r\leq n\right\}$.**7** **Step 5:** Try to utilize $sc_{r}\left(1\leq r\leq n\right)$ to recover a secret pixel position of $S_{2}$ denoted by $s_{2}\left(w_{2},h_{2}\right)\in \left\{s\left(w_{2},h_{2}\right)|1\leq w_{2}\leq W_{2},1\leq h_{2}\leq H_{2}\right\}$. If successful, return to Step 2 and operate at the next segment; otherwise, return to Step 3.**8** **Step 6:** Utilize all $\lceil \frac{8}{m}\rceil \times n$ shadow pixels $sc_{jr}$ of each segment to compose shadow images $SC_{r}\left(w_{1},h_{1}\right)\in \left\{SC_{r}\left(w_{1},h_{1}\right)|1\leq r\leq n,1\leq w_{1}\leq W_{1},1\leq h_{1}\leq H_{1}\right\}$.**9** **Step 7:** Output *n* shadow images $SC_{r}\left(1\leq r\leq n\right)$ with a size of $W_{1}\times H_{1}$.



Regarding Algorithm 2, there are several comments as follows:

1. The meaning of the input legal secret images is that we should guarantee these secret images can be appropriately or perfectly applied in (n,n)-threshold SIS, where a concrete (n,n)-threshold SIS limits the conditions on secret images. For example, if the input SIS only applies to grayscale secret images, both secret images should be grayscale.
2. The size relation between the primary secret image and the secondary one is $W_{1}\times H_{1}\geq W_{2}\times H_{2}\times m$ so that we can hide all shadow images of the secondary secret image into those of the primary one. Possibly, there are several pixels in shadows of the primary secret image, which are free from being screened and irrelevant to the secondary secret image.
3. By lots of experiments, *m* in Algorithm 2 are suggested as 1, 2, 3 and 4, which considers the balance among time, hidden capability and other users’ demands. For example, if users pay more attention to hidden capability rather than the average time, more bits in the front positions can be considered as the payload. More experimental results are presented in Section 5.
4. The designed random elements utilization model in step 3 can generate shadow pixels, which is a general model and applicable for different SISSs.
5. Although we design the size of two secret images, the embedding capacity of Algorithm 2 with *m*-bit payload as m=1 is at least the same as another scheme for multiple secrets [22]. The larger *m*, the higher the embedding capacity, which will be validated in Section 4 and Section 5.

**Algorithm 3:** The Recovery of the Proposed (n,n)-threshold IHSD-MSISS.**1** **Input:***n* shadow images $SC_{r}\left(1\leq r\leq n\right)$ with a size of $W_{1}\times H_{1}$; the SIS applied in Algorithm 1.**2** **Output:** the secret image $S_{1}$ with a size of $W_{1}\times H_{1}$; the secret image $S_{2}$ with a size of $W_{2}\times H_{2}$.**3** **Step 1:** For each shadow image $SC_{r}\in \left\{SC_{r}|1\leq r\leq n\right\}$, each time fetch continuous $\left\lceil \frac{8}{m}\right\rceil$ shadow pixels until the last one, which are as the rith segment denoted by $pc_{ri}$. There are $\left\lceil \frac{W_{1}\times H_{1}}{\left\lceil \frac{8}{m}\right\rceil }\right\rceil$ segments in $SC_{r}$. Then, repeat Step 2.**4** **Step 2:** For each segment $pc_{ri}\in \left\{pc_{ri}|1\leq r\leq n,1\leq i\leq \left\lceil \frac{W_{1}\times H_{1}}{\left\lceil \frac{8}{m}\right\rceil }\right\rceil \right\}$ of $SC_{r}$, repeat Step 3.**5** **Step 3:** For each pixel $sc_{j}\in \left\{sc_{j}|1\leq j\leq \left\lceil \frac{8}{m}\right\rceil \right\}$ in $pc_{ri}$, extract *m* front bits and link in turn as a shadow pixel, denoted by $sc_{ri}^{\prime }$.**6** **Step 4:** Utilize shadow pixels $sc_{ri}^{\prime }\in \left\{sc_{ri}^{\prime }|1\leq i\leq \left\lceil \frac{W_{1}\times H_{1}}{\left\lceil \frac{8}{m}\right\rceil }\right\rceil \right\}$ to compose shadow images ${SC^{\prime }}_{r}\left(w_{2},h_{2}\right)\in \left\{{SC^{\prime }}_{r}\left(w_{2},h_{2}\right)|1\leq r\leq n,1\leq w_{2}\leq W_{2},1\leq h_{2}\leq H_{2}\right\}$.**7** **Step 5:** Utilize $SC_{r}$ and ${SC^{\prime }}_{r}\left(1\leq r\leq n\right)$ by the determined SIS to recover all secret pixels of $S_{1}$ and $S_{2}$ respectively, which are denoted by $s_{t}\left(w_{t},h_{t}\right)\in \left\{s_{t}\left(w_{t},h_{t}\right)|t\in \left\{1,2\right\},1\leq w_{t}\leq W_{t},1\leq h_{t}\leq H_{t}\right\}$.**8** **Step 6:** Put secret pixels of $S_{1}$ and $S_{2}$ in respective position.**9** **Step 7:** Output the reconstructed secret image $S_{1}$ with a size of $W_{1}\times H_{1}$ and $S_{2}$ with a size of $W_{2}\times H_{2}$.

Regarding Algorithm 3, there are several comments as follows:

1. Algorithm 3 must input the same (n,n)-threshold SIS as that of Algorithm 2. Otherwise, it is invalid for the recovery of two secret images.
2. From *n* shadow images $SC_{r}$, steps 1–4 generate shadow images of $S_{2}$, denoted by $SC_{r}^{\prime }$. Extraction of shadow pixels of $S_{2}$ is inverse to the combination. Thus, we aim at the *r*th shadow image $SC_{r}$ and take *m* front bits of each pixel in arrangement positions of the image to generate $SC_{r}^{\prime }$.
3. Algorithm 3 depicts the concrete operation for recovery. From a general perspective, (n,n)-threshold IHSD-MSISS utilizes *n* shadow images $SC_{r}$ to reconstruct two secret images simultaneously. Indeed, Algorithm 3 generates $SC_{r}^{\prime }$ from $SC_{r}$ so that each of two secret images can be reconstructed individually, which is more flexible and efficient.

### 3.2. Evaluating Metrics

1. **Shadow image randomness.**(n,n)-treshold IHSD-MSISS simultaneously shares two secret images. It is necessary for shadow images to be noise-like by the naked eye. In addition, shadow images can be evaluated in a histogram plot; the more uniform the distribution of the histograms is, the more secure the proposed scheme.
2. **Recovered secret image fidelity.** Fidelity refers to the similarity between the original secret images and the reconstructed images. The metric can be measured by Peak Signal-to-Noise Ratio (PSNR) shown in Equation (4) and Structural Similarity (SSIM) shown in Equations (5) and (6). PSNR evaluates the image similarity, and MSE indicates the mean square error. SSIM is the metric of image structural similarity, where $\mu_{x}$, $\sigma_{x}^{2}$, $\sigma_{xy}$ and *L* denote the average of *x*, the variance of *x*, the covariance of *x* and *y*, and image pixel value range, respectively.
  (4) $$\left\{\begin{array}{c} PSNR=10\times log_{10}\left(\frac{255^{2}}{MSE}\right)dB,\\ MSE=\frac{1}{W\times H}\sum_{i=1}^{W}\sum_{j=1}^{H}{\left[S^{\prime }\left(i,j\right)-S\left(i,j\right)\right]}^{2} \end{array}\right.$$
  (5) $$SSIM\left(S,S^{\prime }\right)=\frac{\left(2\mu_{S}\mu_{S^{\prime }}+c_{1}\right)\left(2\sigma_{SS^{\prime }}+c_{2}\right)}{\left(\mu_{S}^{2}+\mu_{S^{\prime }}^{2}+c_{1}\right)\left(\sigma_{S}^{2}+\sigma_{S^{\prime }}^{2}+c_{2}\right)}$$
  (6) $$\left\{\begin{array}{c} c_{1}={\left(0.01\times L\right)}^{2},\;c_{2}={\left(0.03\times L\right)}^{2},\\ \left\{x,y|S,S^{\prime }\;\mathrm{and}\;x\neq y\right\},\;\left\{L|0\leq L\leq 255\right\},\\ \mu_{x}=\frac{1}{W\times H}\sum_{i=1}^{W}\sum_{j=1}^{H}x\left(i,j\right),\;\sigma_{x}=\sqrt{\sigma_{x}^{2}},\\ \sigma_{x}^{2}=\frac{1}{W\times H-1}\sum_{i=1}^{W}\sum_{j=1}^{H}{\left(x\left(i,j\right)-\mu_{x}\right)}^{2},\\ \sigma_{xy}=\frac{1}{W\times H-1}\sum_{i=1}^{W}\sum_{j=1}^{H}\left(x\left(i,j\right)-\mu_{x}\right)\left(y\left(i,j\right)-\mu_{y}\right) \end{array}\right.$$
3. **The embedding rate.** According to the relative importance of two secret images, the embedding rate of (n,n)-threshold IHSD-MSISS denoted by ER is defined as the average secret information bit per share bit and evaluated in Equation (7), where $V_{x}$, $W_{x}$, $H_{x}$, $L_{x}$ and *n* denote the weight factor, image width, image height, grayscale level and number of *x*, respectively. *x* can be set as *i* or SC, which describe that the *i*th secret image or shadow image (SC). $L_{x}=8$ means that *x* is a grayscale image, while $L_{x}=1$ represents a binary image. The larger ER, the higher the embedding rate.
  (7) $$ER=\frac{V_{1}\times W_{1}\times H_{1}\times L_{1}+V_{2}\times W_{2}\times H_{2}\times L_{2}}{n\times W_{SC}\times H_{SC}\times L_{SC}}$$
4. **Average time and the quantity of available random pixels.** Average time of (n,n)-threshold IHSD-MSISS can be divided as average sharing time and average recovery time. The less time, the more efficiency the proposed scheme. Besides average time, we add the quantity of available random pixels for a more intuitive evaluation.
  The quantity of available random pixels denoted by *q* is defined as the total number of qualified shadow pixels in a single sharing phase of a primary secret pixel. The combination from front bits of qualified shadow pixels is sure to recover the pixel of the secondary secret image, and a successful try for recovery just needs *n* qualified shadow pixels. Thus, in a single sharing phase, the quantity of available random pixels measures the total number of qualified shadow pixels of a primary secret pixel, while it is in the same way for other primary secret pixels. The equation of *q* relies on the SIS input.
  SIS provides many available random elements in the sharing phase. By controlling and screening the randomness of SIS, we proposed the (n,n)-threshold IHSD-MSISS. Thus, the quantity of available random pixels is a valid metric to measure the efficiency. The larger the quantity, the greater the possibility of qualified combinations, along with fewer failed tries. Therefore, the time will be less.

### 3.3. An Example

Here, we assume that a user applies PSISS to the proposed (n,n)-threshold IHSD-MSISS ((n,n)-threshold IHSD-PMSISS) and chooses 1-bit payload. A grayscale image of 256×256 is as the primary secret image, while a grayscale image of 128×64 is as the secondary secret image.

**Figure 4:**
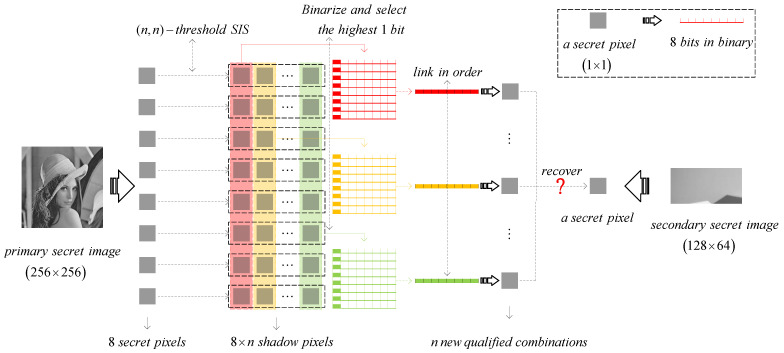
The diagram of 1-bit screened (n,n)-threshold IHSD-PMSISS.

Owing to 1-bit payload, the proposed scheme aims at 8 pixels of the primary secret image. During the sharing phase, each pixel is divided into *n* shadow pixel. There are 8×n shadow pixels in total. Then, we extract the highest 1 bit from the first shadow pixel of the first secret pixel, and continue to extract the highest 1 bit from the first shadow pixel of the second secret pixel until the first shadow pixel of the eighth secret pixel. These 8 payload bits will be combined in order as the first combination. Similarly, we select the highest 1 bit from the second, third, ⋯, *i*th, ⋯, until the *n*th shadow pixels from the first secret pixel to the eighth one, and combine them into the *i*th (i=2,⋯,n) combination. By *n* new comparisons, we try to recover a secondary secret pixel. The corresponding diagram is shown as Figure 4.

In this example, the embedding rate denoted by $ER_{1-bit}^{example}$ is calculated in Equation (8). The quantity of available random pixels denoted by $q_{1-bit}^{example}$ is shown as Equation (9), which will be proved in Section 4.

(8) $$ER_{1-bit}^{example}=\frac{V_{1}\times 256\times 256\times 8+V_{2}\times 128\times 64\times 8}{n\times 256\times 256\times 8}=\frac{8V_{1}+V_{2}}{8n}$$

(9) $$q_{1-bit}^{example}=\frac{p^{n-1}}{2^{\left(m\times n\right)}}=\frac{p^{n-1}}{2^{\left(1\times n\right)}}=\frac{p^{n-1}}{2^{n}}$$

### 3.4. Highlights and Comments

1. **Highlights**. The (n,n)-threshold IHSD-MSISS fuses the sharing phase of the secondary secret image into the sharing phase of the primary secret image, which is an undivided process to share two secret images simultaneously. By controlling the randomness of SIS, the proposed scheme generates shadow images of the primary secret image. Several front bits of pixels in shadow images can be combined as the shadow images which can recover the secondary secret image. After the sharing phase of the proposed scheme, we can obtain *n* shadow images, and they can reconstruct two secret images simultaneously in the recovery phase.
  The strengths and weaknesses of (n,n)-threshold IHSD-MSISS.
2. **Strengths**.
  - (n,n)-threshold IHSD-MSISS is general, applicable and undivided.
  - (n,n)-threshold IHSD-MSISS is in low complexity, which can recover secret images losslessly and improve the efficiency of sharing multiple secrets (such as saving the storage and improving the embedding capacity of key information).
  - (n,n)-threshold IHSD-MSISS promotes the capability to resist tampering and strengthen the security.
3. **Weaknesses**. The effect of (n,n)-threshold IHSD-MSISS strongly relies on inputs of a concrete SISS and secret images, influencing the average time and recovery quality of multiple secret images.

## 4. Theoretical Analyses and Proof

In this section, the proposed (n,n)-threshold IHSD-MSISS is analyzed in theory. We apply a PSIS scheme (PSISS) to the proposed scheme, that is (n,n)-threshold IHSD-PMSISS, and use the example to discuss clearly and thoroughly. In addition, evaluating metrics of the proposed scheme are discussed. Especially, the embedding rate ER and the quantity of available random pixels *q* are theoretically demonstrated for their validity and objectivity.

**Analysis** **1.** *The proposed (n,n)-threshold IHSD-MSISS is undivided and perfectly feasible in theory.*

**Proof.**  (n,n)-threshold IHSD-MSISS is whole and undivided based on IHSD [25]. SIS of various principles can create random elements, and secret images by SIS will be shared into noise-like shadow images. We focus on the randomness of SIS. By the designed random elements utilization model, we control and screen among the random elements generated in the sharing phase. At the beginning, we generate *n* shadow images from the primary secret image. During the sharing phase, we select *m* bits from shadow pixels as the payload, and we combine them in a designed order. Then, we utilize the new combinations and try to recover a pixel of the secondary secret image. If the recovery is failed, we will adopt the random elements utilization model again to create lots of random elements for new combinations and tries, so that it is greatly possible for the new combination to recover the secondary secret pixel. Thus, the proposed scheme is perfectly feasible in theory.

In addition, the information hiding condition of IHSD must require the reversibility, while the proposed scheme utilize SIS into IHSD to fuse the sharing phase of the secondary secret image into the sharing phase of the primary secret image. SIS is reversible, so the proposed scheme can realize by IHSD.

Moreover, the sharing phase of the proposed scheme is the sharing phase of the primary secret image with other operations. After the sharing phase, *n* shadow images carries the information of two secret images. So the proposed scheme is an undivided scheme for sharing multiple secrets. □

**Analysis** **2.** *(n,n)-threshold IHSD-MSISS is secure in theory.*

**Proof.**  (n,n)-threshold IHSD-MSISS applies the idea of IHSD [25], which describes that the sharing phase of SIS not only can protect a secret image but also can hide extra information at the same time. An SIS generates many random pixels for a secret image to confuse images’ original features. To avoid suspicion and maintain enough randomness, hidden information must be absolutely random in IHSD for promoted security. Nature images are born with texture features. By SIS, the images will be divided as noise-like shadow images. From this idea, the proposed scheme realizes by utilizing SIS in IHSD. It fuses the sharing phase of the secondary secret image into the sharing phase of the primary secret image. The shadow images of the secondary secret can be extracted from the shadow images of the primary secret, which relies on SIS. Thus, the proposed scheme is safe. Moreover, the histograms of final shadow images should be well-distributed theoretically. □

**Analysis** **3.** *(n,n)-threshold IHSD-MSISS is general and applicable.*

**Proof.**  Existent popular SIS including PSIS, CRTSIS and VC can generate lots of random elements in the sharing phase. From the principle, we proposed the (n,n)-threshold IHSD-MSISS. Thus, one of the above SISS with the proper images can be applied in the proposed scheme. □

**Analysis** **4.** *(n,n)-threshold IHSD-MSISS shares two secret images into n shadow images. In the recovery phase, n shadow images can recover both secret images simultaneously, so that the recovery rate is 100%. Moreover, if any shadow images are missed or damaged, neither secret image will be recovered.*

**Proof.**  (n,n)-threshold IHSD-MSISS relies on (k,n)-threshold SIS. When k=n, the number of shadow images participated in the sharing and recovery phase is the same. So, in the recovery phase, by *n* shadow images we can obtain two secret images without loss, and the recovery rate is 100%.

In addition, the proposed scheme realizes based on the sharing phase of the primary secret image. From *n* shadow images of the primary secret image, we can extract *n* shadow images of the secondary secret image. *n* shadow images generated from the proposed scheme carries the information of two secret images. Thus, any lack or modification of shadow images will fail recovery for both secret images. □

**Analysis** **5.** *Recovered secret image fidelity relies on the concrete inputs in (n,n)-threshold IHSD-MSISS. By the proposed scheme, the secret images can be recovered losslessly. The fidelity is independent of the realization principle of the proposed scheme.*

**Proof.**  Recovered secret image fidelity of (n,n)-threshold IHSD-MSISS relies on the user’s inputs, which is relevant with pixel values of the secret images and concrete SIS parameters.

For instance, we use PSISS with prime p=251 and share two secret images whose pixels both range from 0 to 250. The recovered images will be lossless. However, in the case of PSISS with p=251 and secret images with [0,255], we will obtain two recovered secret images with loss. □

**Analysis** **6.** *In (n,n)-threshold IHSD-MSISS, the embedding rate ER is defined as Equation (7).*

**Proof.**  In (n,n)-threshold IHSD-MSISS, we aim at two secret images in the sharing phase, each in grayscale level $L_{i}$ with the size of $W_{i}\times H_{i}$ and the weight factor $V_{i}$. Then, we get *n* shadow images of grayscale level $L_{SC}$ with the size of $W_{SC}\times H_{SC}$. The proposed scheme protect two secret images by *n* shadow images. Thus, the equation of embedding rate ER, which measures the rate of each share bit carrying with secret images, can be calculated in Equation (7). By the equation, we also know that the larger (n,n)-threshold, the less ER. □

**Analysis** **7.** *The quantity of available random pixels denoted by q is the total number of qualified shadow pixels in a single sharing phase of a primary secret pixel. q has to be calculated by a concrete SISS. By (n,n)-threshold IHSD-PMSISS, q will be calculated in Equation (10), where p, m and n denote prime p of PSISS, the number of payload bits and (n,n)-threshold, respectively.*

(10) $$q^{IHSD-PMSISS}=\frac{p^{n-1}}{2^{m\times n}}$$

**Proof.**  In (n,n)-threshold IHSD-PMSISS, a primary secret pixel is shared by a random (n−1)-degree polynomial with *n* coefficients denoted by $a_{0}$, $a_{1}$, ⋯, $a_{n}$. Except $a_{0}$, the other coefficients range from 0 to p−1. There are *p* possibilities for each coefficient so that a random polynomial has $p^{n-1}$ possibilities. Once the polynomial is determined, *n* shadow images generated in the sharing phase will be decided. For a primary secret pixel, there are *n* shadow pixels generated by a random polynomial, which has $p^{n-1}$ probabilities. Users need to decide the number of payload bits, that is *m*-bit payload, in (n,n)-threshold IHSD-PMSISS, where *m*-bit payload means selecting *m* front bits from each shadow pixel (*n* shadow pixels in total) out of the primary secret pixel. Eight bits of shadow pixels are 0 or 1 in binary. So, there are two possibilities for each payload bit. Among $p^{n-1}$ possibilities, *m*-bit payload in *n* shadow pixels should be considered together for judgment of qualified shadow pixels, which has $2^{m\times n}$ possibilities. Thus, the equation of *q* in (n,n)-threshold IHSD-PMSISS is calculated in Equation (10). Moreover, the quantity of available random pixels of the example in Section 3, denoted by $q_{1-bit}^{example}$, can be also proved as Equation (9). □

**Analysis** **8.** *The sharing and recovery time of (n,n)-threshold IHSD-MSISS is dependent on the embedding rate and the quantity of available random pixels, where we conclude theoretically that the higher embedding rate, the more the time, while the greater the quantity of available random pixels, the less the time.*

**Proof.**  According to Algorithm 1, *m*-bit payload means that we simultaneously share $\left\lceil \frac{8}{m}\right\rceil$ secret pixels. In (n,n)-threshold IHSD-MSISS, the higher embedding rate implies that the payload bits are more (*m* is larger). It is more difficult to combine from shadow pixels of fewer secret pixels ($\left\lceil \frac{8}{m}\right\rceil$ is fewer), which will take more time. In addition, as the quantity of available random pixels is more, the probability of combinations from qualified shadow images will be higher, so the proposed scheme will spend less time sharing multiple secrets. □

## 5. Experimental Results and Comparisons

In this section, we show the experimental results of (n,n)-threshold IHSD-PMSISS which are discussed in detail in 1-bit, 2-bit and 4-bit payload. Then, we experiment and verify on parameters analyzed theoretically in Section 4. Some discussions are given. Finally, comparisons with the scheme for multiple secrets introduced in [22] are demonstrated for the strengths of the proposed scheme.

All experiments in this paper are demonstrated on the computer of Intel(R)Xeon (R CPUE5-2630v4@220 GHz 2.20 GHz, where RAM’ 64.0 GB (63.9 GB available) and operating system is 64-bit for x64 processors.

### 5.1. Illustration

**Figure 5:**
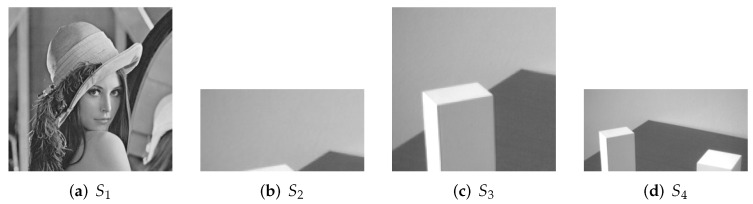
Experimental images. (**a**) Grayscale primary secret image of size 256 × 256; (**b**) grayscale secondary secret image of size 128 × 64; (**c**) grayscale secondary secret image of size 128 × 128; (**d**) grayscale secondary secret image of size 256 × 128.

All secret images for experiments are presented in Figure 5, which are all grayscale images. We choose (a) with the size of 256×256 as the primary secret image. (b) of 128×64, (c) of 128×128 and (d) of 256×128 are considered as the secondary secret images, which are applied in 1-bit, 2-bit and 4-bit payload respectively.

**Figure 6:**
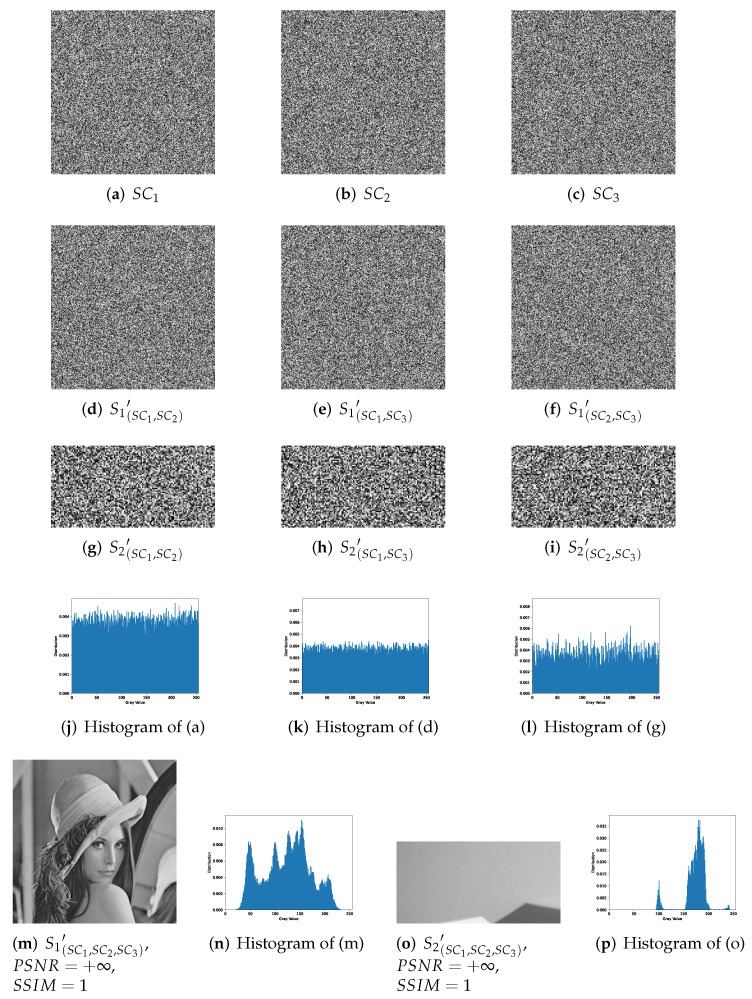
Experimental results of the proposed 1-bit payload IHSD-PMSISS in (3,3)-threshold. (**a**–**c**) Three grayscale shadow images $SC_{1}$, $SC_{2}$ and $SC_{3}$; (**d**–**f**) recovered grayscale primary secret image ${S_{1}}^{\prime }$ with two shadow images; (**g**–**i**) recovered grayscale secondary secret image ${S_{2}}^{\prime }$ with two shadow images; (**j**) the histogram of $SC_{1}$; (**k**) the histogram of ${S_{1}}_{\left(SC_{1},SC_{2}\right)}^{\prime }$; (**l**) the histogram of ${S_{2}}_{\left(SC_{1},SC_{2}\right)}^{\prime }$; (**m**) recovered grayscale primary secret image ${S_{1}}^{\prime }$ with all shadow images; (**n**) the histogram of ${S_{1}}_{\left(SC_{1},SC_{2},SC_{3}\right)}^{\prime }$; (**o**) recovered grayscale secondary secret image ${S_{2}}^{\prime }$ with all shadow images; (**p**) the histogram of ${S_{2}}_{\left(SC_{1},SC_{2},SC_{3}\right)}^{\prime }$.

Figure 6 shows a complete experimental result of 1-bit payload IHSD-PMSISS in (3,3)-threshold. Here, Figure 5a $S_{1}$ is the primary secret image, while Figure 5b $S_{2}$ is the secondary secret image. In Figure 6, (a)–(c) denoted by $SC_{1}$, $SC_{2}$ and $SC_{3}$ are noise-like grayscale shadow images generated in the sharing phase. Their size same as the primary secret, is 256 × 256. In the recovery phase, a group of any two shares is calculated to generate reconstructed secrets. The results are listed in (d)–(i), which are still noise-like without anything distinguishable. We demonstrate the histogram distributions from (a) to (i), and one result of the respective case is presented in (j)–(l). (j) is the histogram of shadow image $SC_{1}$. (k) and (l) are the histograms of recovered secret images by $SC_{1}$ and $SC_{2}$. (m) and (o) describe the results reconstructed with all shares, where (n) and (p) are the histograms of (m) and (o), respectively. The metrics of PSNR and SSIM are listed in the respective image label.

**Figure 7:**
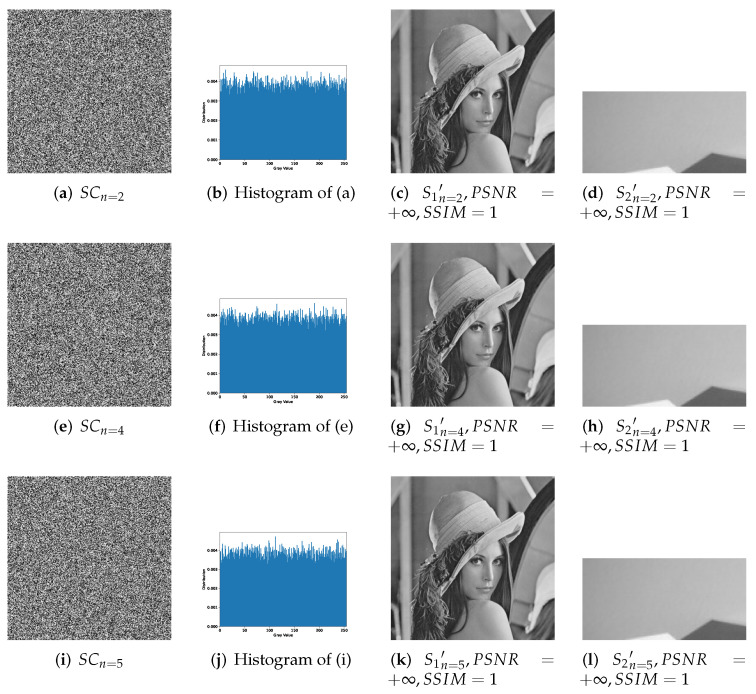
Experimental results of the proposed 1-bit payload IHSD-PMSISS in (n,n)-threshold where n=2,4,5. (**a**–**d**) Results of n=2; (**e**–**h**) results of n=4; (**i**–**l**) results of n=5.

**Figure 8:**
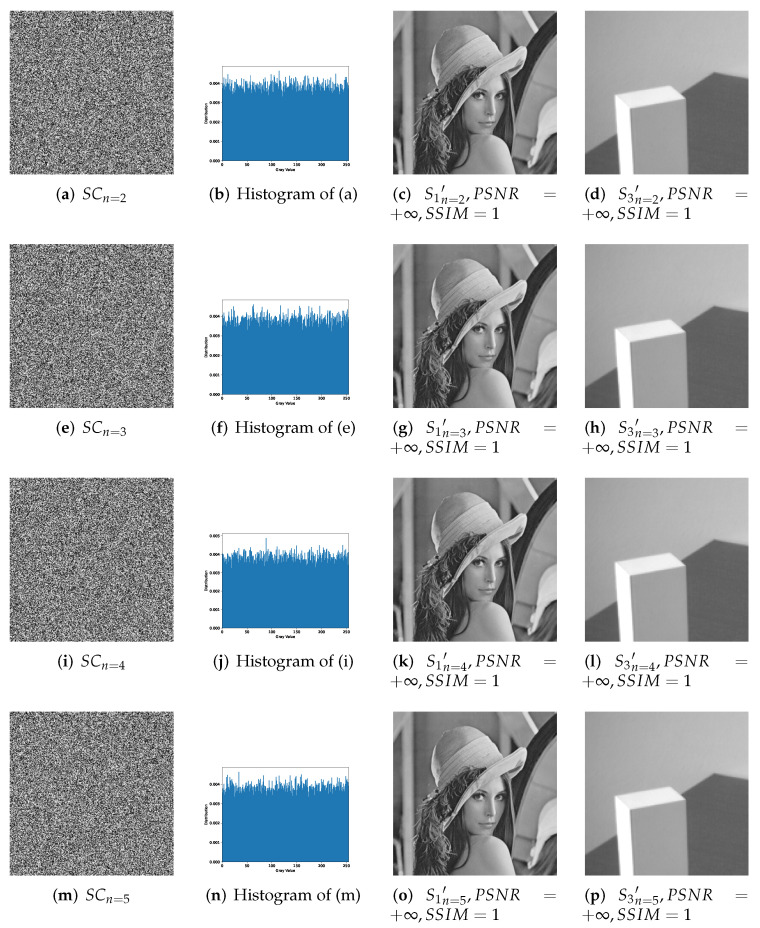
Experimental results of the proposed 2-bit payload IHSD-PMSISS in (n,n)-threshold where n=2,3,4,5. (**a**–**d**) Results of n=2; (**e**–**h**) results of n=3; (**i**–**l**) results of n=4; (**m**–**p**) results of n=5.

**Figure 9:**
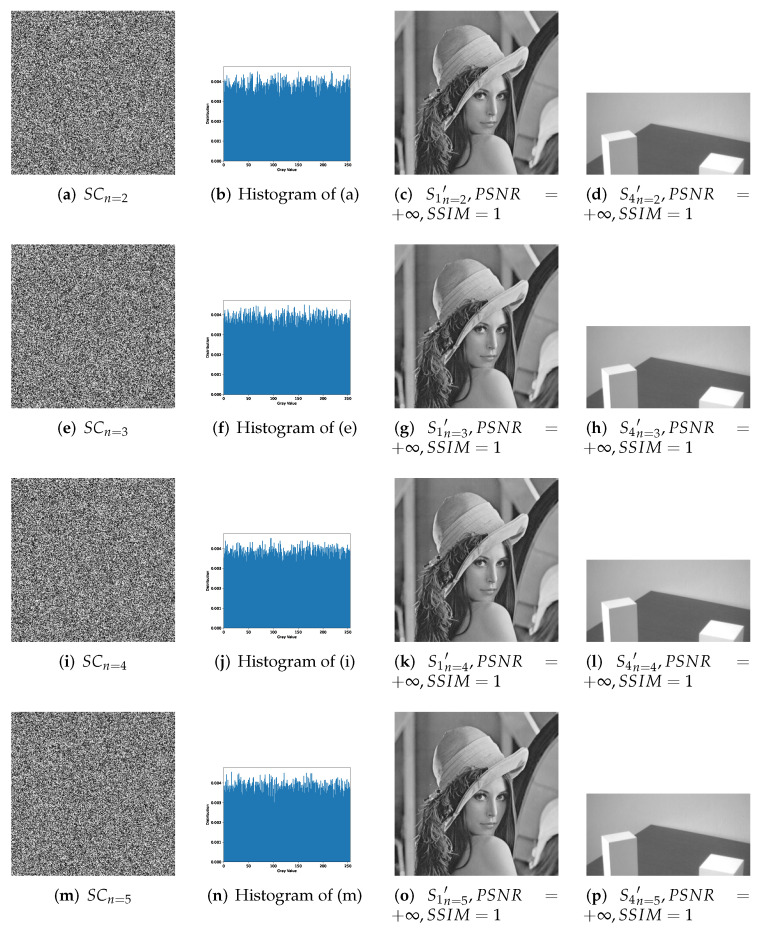
Experimental results of the proposed 4-bit payload IHSD-PMSISS in (n,n)-threshold where n=2,3,4,5. (**a**–**d**) Results of n=2; (**e**–**h**) results of n=3; (**i**–**l**) results of n=4; (**m**–**p**) results of n=5.

Figure 7, Figure 8 and Figure 9 effectively confirm the feasibility and security of (n,n)-threshold IHSD-PMSISS. We select Figure 5a $S_{1}$ as the primary secret. Figure 5b $S_{2}$, Figure 5c $S_{3}$, and Figure 5d $S_{4}$ are the secondary secrets in experiments. Corresponding results are shown in Figure 7, Figure 8 and Figure 9, respectively.

Figure 7 presents the experimental results of 1-bit payload, where (a)–(d), (e)–(h) and (i)–(l) describe the experiments of (n,n)-threshold as n=2,4,5 severally. In Figure 7, (a), (e) and (i) are one of *n* grayscale shadow images. (b), (f) and (j) are the corresponding histograms of (a), (e) and (i). (c), (g) and (k) are recovered primary secrets in respective threshold, while (d), (h) and (l) are reconstructed secondary secret. All images generated in the recovery phase are labeled with their PSNRs and SSIMs. Figure 8 and Figure 9 show the experimental results of n=2,3,4,5 and illustrate the process of 2-bit payload and 4-bit payload separately, which are in the same case in Figure 7.

Based on the above illustrations, we can summarize the following:

1. (n,n)-threshold IHSD-PMSISS referred in Section 4 has been experimented. We discuss and list part experimental results where *n* is from 2 to 5.
2. All shadow images generated in the sharing phase of (n,n)-threshold IHSD-PMSISS are noise-like. The corresponding histograms have a uniform distribution, which indicates these shares are without any leakage of both secret images.
3. In Figure 6, with the limitation of (3,3)-threshold, only by three shadow images can two secret images be recovered. Otherwise, there is nothing about the content of any secret. The presented histograms depict that the recovered results by two shares reveal nothing about secrets. In addition, both recovered secret images are identical with relative original secret images as PSNR=+∞ and SSIM=1.
4. In (n,n)-threshold IHSD-PMSISS, we can share two secret images in the sharing phase. In the recovery phase, two secret images can be recovered losslessly at the same time. Thus, we can conclude that not only (n,n)-threshold IHSD-PMSISS, but also the general IHSD-MSISS is available and effective, which confirms the theories in Section 4 forcefully.

### 5.2. Relevant Parameters and Analyses

We still take (n,n)-threshold IHSD-PMSISS of 1-bit, 2-bit and 4-bit payload and experiment on the following vital parameters, namely, the embedding rate, average time and the quantity of available random pixels. Concrete experimental results of parameters are analyzed in detail and presented through tables and curves.

#### 5.2.1. The Embedding Rate

The definition of the embedding rate denoted by ER has been mentioned in Section 3 and proved in Section 4, and we have discussed three payload situations of (n,n)-threshold IHSD-PMSISS theoretically. Although the size of two secret images in each situation has been set, the embedding rate is the most objective metric to measure the payload. With the above illustration of experimental images, the embedding rate in each situation can be calculated by Equation (7).

**Figure 10:**
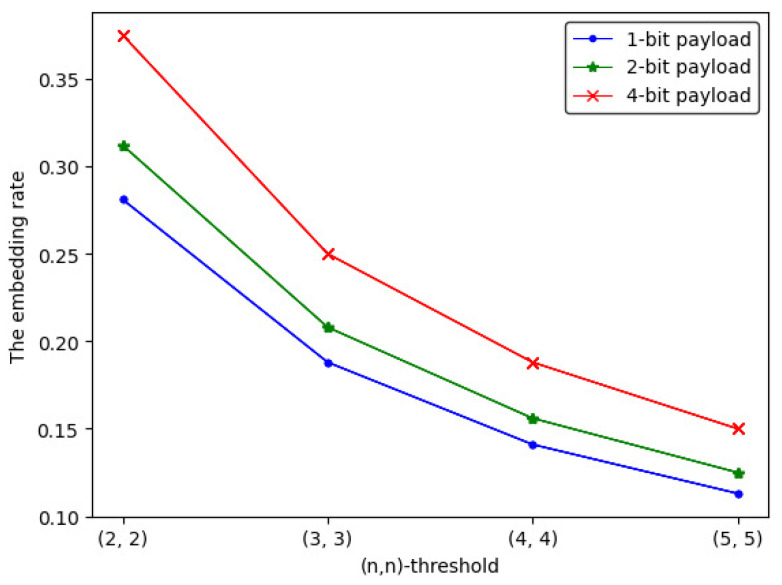
The embedding rate curve of (n,n)-threshold IHSD-PMSISS in three payload situations.

Here, we assume that two secret images have the same degree of importance. The embedding rate of 1-bit, 2-bit and 4-bit payload (n,n)-threshold IHSD-PMSISS is shown as Equation (11). When *n* is from 2 to 5, the embedding rate is listed in Table 1, whose trend is depicted in Figure 10.

(11) $$\begin{array}{cc} ER_{1-bit} & =\frac{\frac{1}{2}\times 256\times 256\times 8+\frac{1}{2}\times 128\times 64\times 8}{n\times 256\times 256\times 8}=\frac{9}{16\times n}\\ ER_{2-bit} & =\frac{\frac{1}{2}\times 256\times 256\times 8+\frac{1}{2}\times 128\times 128\times 8}{n\times 256\times 256\times 8}=\frac{5}{8\times n}\\ ER_{4-bit} & =\frac{\frac{1}{2}\times 256\times 256\times 8+\frac{1}{2}\times 256\times 128\times 8}{n\times 256\times 256\times 8}=\frac{3}{4\times n} \end{array}$$

The corresponding curve shown in Figure 10 benefits us to observe the trend of ER more intuitively, from which we can find that, as *n* is increasing, $ER_{i}\left(i=1,2,3\right)$ presents a downward trend. Among three payload situations, the embedding rate of 4-bit payload is the largest.

#### 5.2.2. Average Time

**Figure 11:**
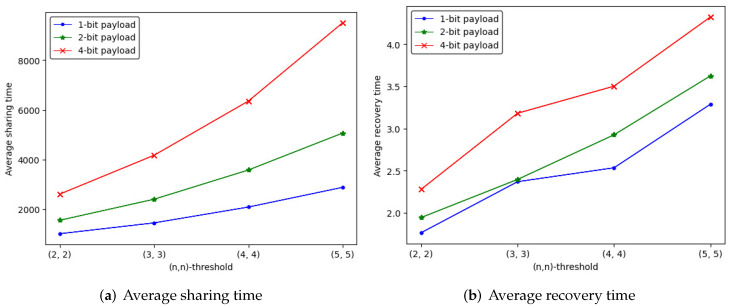
The average time curve of (n,n)-threshold IHSD-PMSISS in three payload situations.

We compute the sharing and recovery time spent on (n,n)-threshold IHSD-PMSISS of three payload situations. The average sharing and recovery time is listed in Table 2 and Table 3, where the trend is depicted in Figure 11.

Different from the trend of the embedding rate, the average time is increases as *n* grows. The 4-bit payload takes more time for sharing and recovery. Through many experiments, we find that the experimental deviation of average sharing time ranges within two minutes up and down, and that of average recovery time varies within two seconds.

#### 5.2.3. The Quantity of Available Random Pixels

In (n,n)-threshold IHSD-PMSISS, random pixels utilized for a set of shadows with two secret images are produced from PMSISS, where *p* is valued as 257 for lossless recovery. Relevant theories and Equation (10) have been mentioned in Section 4. The quantity of available random pixels of 1-bit, 2-bit and 4-bit payload are counted by $q_{1}$, $q_{2}$ and $q_{3}$ in Equation (12), respectively. The concrete results are listed in Table 4.

(12) $$q_{1}=\frac{p^{n-1}}{2^{n}},\;q_{2}=\frac{p^{n-1}}{2^{2\times n}}=\frac{p^{n-1}}{4^{n}},\;q_{3}=\frac{p^{n-1}}{2^{4\times n}}=\frac{p^{n-1}}{16^{n}}$$

**Figure 12:**
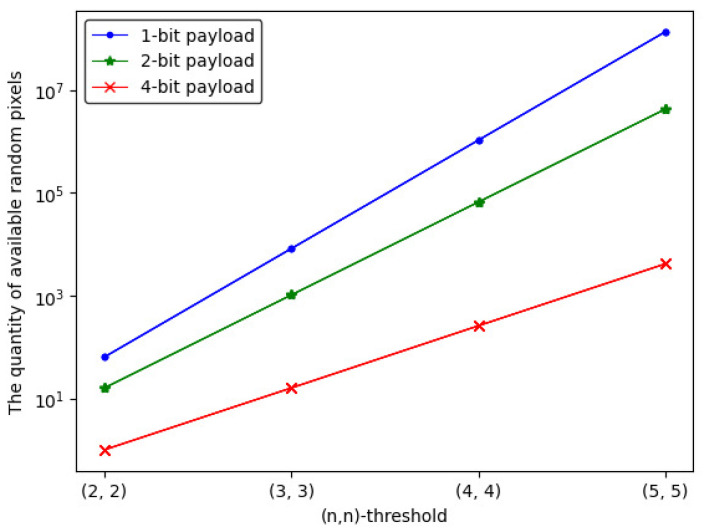
The quantity curve of available random pixels of (n,n)-threshold IHSD-PMSISS in three payload situations.

Figure 12 describes the trend of the quantity curve of available random pixels, from which we can notice that the whole tendency is ascending with *n* increases. The 1-bit payload has more available quantities than the others.

#### 5.2.4. Relevance of Above Parameters

The above vital parameters have been studied with verification, and we discuss their relevance. With experimental results and curves, we can draw the following conclusions:

1. In Figure 10 and Figure 11, the embedding rate of 4-bit payload is higher than the others, which spends the most average time on sharing and recovery. The embedding rate is proportional to the average time for the same (n,n)-threshold.
2. In Figure 11 and Figure 12, the quantity of available random pixels of 1-bit payload is the largest, while 1-bit payload takes less time to share and recover than the others. For the same (n,n)-threshold, the less the quantity of available random pixels, the more the average sharing and recovery time.

### 5.3. Comparisons with the Scheme Proposed by Liu et al.

We will compare our proposed (n,n)-threshold IHSD-MSISS with the scheme of Liu et al. [22], which also achieves a MSISS. Liu et al. combine RGVCS and PSISS to propose an ideal $\left(k_{1},k_{2},n\right)$-threshold TiOSISS for multiple secrets, where $\left(k_{1},n\right)$-threshold is for RGVCS and $\left(k_{2},n\right)$-threshold is for PSISS. For comparison, we will demonstrate the experiment with proper parameters to realize the identical results with those of Liu et al.

#### 5.3.1. Illustration Comparison

**Figure 13:**
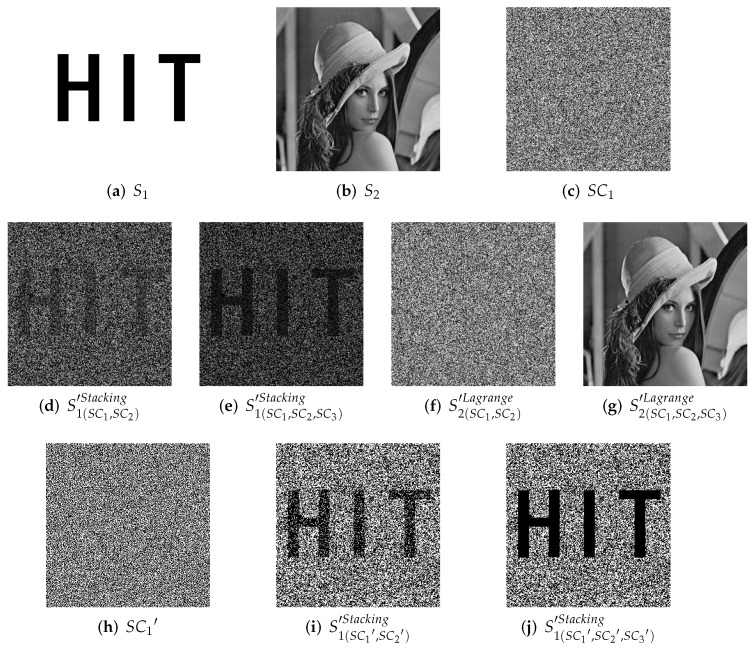
Experimental results of (2,3,3)-threshold TiOSISS scheme. (**a**) Binary secret image; (**b**) grayscale secret image; (**c**) grayscale shadow image $SC_{1}$; (**d**) recovered binary secret image with $SC_{1}$ and $SC_{2}$ in (2,3)-threshold RGVCS; (**e**) recovered binary secret image with all grayscale shadow images in (2,3)-threshold RGVCS; (**f**) recovered grayscale secret image with $SC_{1}$ and $SC_{2}$ in (3,3)-threshold PSISS; (**g**) recovered grayscale secret image with all grayscale shadow images in (3,3)-threshold PSISS; (**h**) binary shadow image generated from $SC_{1}$; (**i**) recovered binary secret image with ${SC_{1}}^{\prime }$ and ${SC_{2}}^{\prime }$ in (2,3)-threshold RGVCS; (**j**) recovered binary secret image with all binary shadow images in (2,3)-threshold RGVCS.

We choose a (2,3,3)-threshold TiOSISS as the comparison instance from [22] and reappear with identical secret images, whose experimental results are described in Figure 13. Here, (a) is a binary secret image $S_{1}$ utilized in (2,3)-threshold RGVCS, while (b) is a grayscale secret image $S_{2}$ for (3,3)-threshold PSISS. One of three grayscale shadow images $SC_{1}$ is listed in (c). Any two of three shadow images can recover the secret image shared in (2,3)-threshold RGVCS. (d) is the result recovered by $SC_{1}$ and $SC_{2}$, while stacking all shares for recovery will have a better visual quality as (e). (f) and (g) show the results in the recovery phase of (3,3)-threshold PSISS. Only by all shadow images can a secret image be reconstructed losslessly. With fewer than three shares, there is nothing about the secret image. Same as Liu et al., we binarize the grayscale shadow images $SC_{i}\left(i=1,2,3\right)$ and one of the results $SC_{1}^{\prime }$ is presented in (h). Then, we stack two random or more binary shadow images for recovery of RGVCS, whose results are much clearer and listed in (i) and (j). All images in Figure 13 are of size 256 × 256.

**Figure 14:**
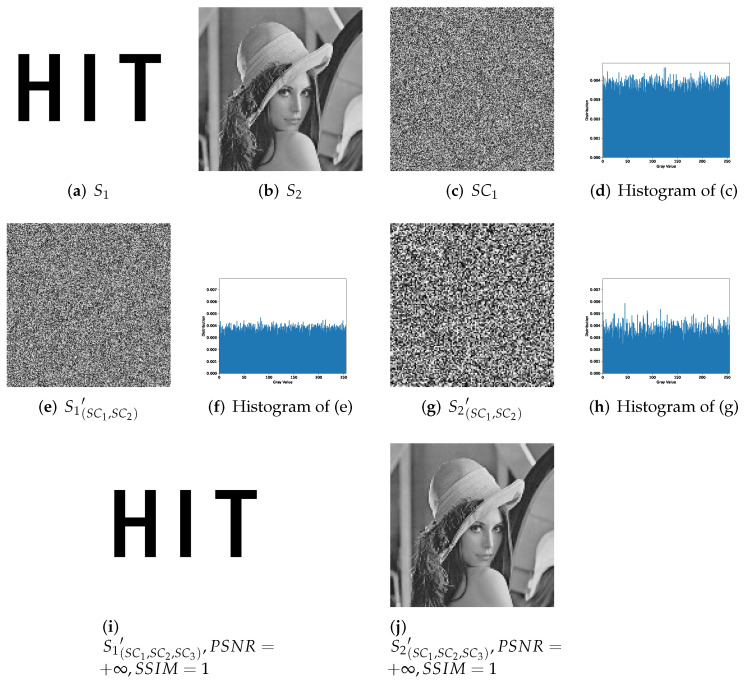
Experimental results of 2-bit payload (3,3)-threshold IHSD-PMSISS. (**a**) Binary secret image of size 256 × 256; (**b**) grayscale secret image of size 128 × 128; (**c**) grayscale shadow image $SC_{1}$ of size 256 × 256; (**d**) the histogram of $SC_{1}$; (**e**) recovered binary secret image ${S_{1}}^{\prime }$ with $SC_{1}$ and $SC_{2}$; (**f**) the histogram of ${S_{1}}_{(SC_{1},SC_{2}}^{\prime })$; (**g**) recovered grayscale secret image ${S_{2}}^{\prime }$ with $SC_{1}$ and $SC_{2}$; (**h**) the histogram of ${S_{2}}_{(SC_{1},SC_{2}}^{\prime })$; (**i**) recovered binary secret image ${S_{1}}^{\prime }$ with all grayscale shadow images; (**j**) recovered grayscale secret image ${S_{2}}^{\prime }$ with all grayscale shadow images.

With secret images of the same content, we experiment one example of our proposed (n,n)-threshold IHSD-MSISS, 2-bit payload (3,3)-threshold IHSD-PMSISS, for comparison. The corresponding results are listed in Figure 14, where (a) is the same binary secret image $S_{1}$ as Figure 13a, and (b) is a grayscale secret image $S_{2}$ of size 128 × 128 with the same content as Figure 13b. Among others in Figure 14, (c) is one of three grayscale shadow images $SC_{1}$ with the relevant histogram shown in (d). We experiment with all the combinations of selecting two shadows from the three for recovery. (e) and (g) is one set of these results recovered from $SC_{1}$ and $SC_{2}$. Relevant histograms, respectively shown in (f) and (h), indicate nothing about secret images. With all shadow images, two secret images are reconstructed without loss and presented in (i) and (j), each of which is identical with the respective original secret image as PSNR=+∞ and SSIM=1.

#### 5.3.2. Parameter Comparison

Based on Figure 13 and Figure 14, we compare (2,3,3)-threshold TiOSISS scheme proposed by Liu et al. with our introduced 2-bit payload (3,3)-threshold IHSD-PMSISS. Some conclusions are listed as follows.

1. Both schemes can achieve the sharing of two secret images with one set of shares.
2. Both schemes can complete the recovery for two secret images.
3. In both schemes, shadow images generated in the sharing phase are noise-like, and their histograms have a uniform distribution, which indicates that there is nothing leaked about any original secret image from shadow images.
4. The proposed IHSD-MSISS is available for (n,n)-threshold, while $\left(k_{1},k_{2},n\right)$-threshold TiOSISS scheme by Liu et al. can realize (k,n)-threshold. In (3,3)-threshold IHSD-PMSISS, we get nothing about secrets by two shadow images. In (2,3,3)-threshold TiOSISS scheme, we can utilize two shadow images to recover the binary secret image based on (2,3)-threshold RGVCS. With (3,3)-threshold PSISS, we cannot obtain anything about secret images from the recovered result by two shares.

Then, we focus on several parameters and analyze the comparisons in detail.

1. **Type of secret images.** Our proposed (n,n)-threshold IHSD-MSISS can provide a general choice for secret images’ type. Binary images, grayscale images, and their combination can be input as secret images in our Algorithm 1. Each of two secret images can be selected as the primary secret image, while another secret image will be the secondary secret image to be hidden. However, in $\left(k_{1},k_{2},n\right)$-threshold TiOSISS proposed by Liu et al., one secret image is a binary image. The other is grayscale. According to the algorithm of Liu et al., it is specific to hide the binary secret image into the grayscale one.
2. **The embedding rate.** The embedding rate of our (n,n)-threshold IHSD-MSISS depends on the concrete SISS and the number of payload bits. The following analyses support three payload situations from (n,n)-threshold IHSD-PMSISS. According to Equation (7), the embedding rate of (n,n)-threshold IHSD-PMSISS in 2-bit payload denoted by $ER_{2-bit}^{IHSD-PMSISS}$ is calculated in Equation (13).
  (13) $$ER_{2-bit}^{IHSD-PMSISS}=\frac{V_{1}\times 256\times 256\times 8+V_{2}\times 128\times 128\times 8}{n\times 256\times 256\times 8}$$
  In $\left(k_{1},k_{2},n\right)$-threshold TiOSISS, the embedding rate is listed in Equation (14), where $V_{x}$, $W_{x}$, $H_{x}$, $L_{x}$ and *n* denote the weight factor, image width, image height, grayscale level and number of *x*, respectively. *x* is *G* (grayscale image) or SC (shadow image) as $L_{x}=8$, while $L_{x}=1$ is for a binary image. The embedding rate of (2,2,3)-threshold TiOSISS is calculated in Equation (15).
  (14) $$ER^{TiOSISS}=\frac{V_{G}\times W_{G}\times H_{G}\times L_{G}+V_{B}\times W_{B}\times H_{B}\times L_{B}}{n\times W_{SC}\times H_{SC}\times L_{SC}}$$
  (15) $$ER_{example}^{TiOSISS}=\frac{V_{1}\times 256\times 256\times 8+V_{2}\times 256\times 256\times 1}{n\times 256\times 256\times 8}$$
  We compare $ER_{2-bit}^{IHSD-PMSISS}$ with $ER_{example}^{TiOSISS}$. By calculation, $ER_{2-bit}^{IHSD-PMSISS}$ is bigger than $ER_{example}^{TiOSISS}$, which means the embedding rate of our proposed scheme is higher than that of Liu et al.’s.
  Moreover, in (n,n)-threshold IHSD-PMSISS of 1-bit and 4-bit payload, the embedding rates denoted by $ER_{1-bit}^{IHSD-PMSISS}$ and $ER_{4-bit}^{IHSD-PMSISS}$ respectively are calculated in Equation (16). We can find that $ER_{1-bit}^{IHSD-PMSISS}$ is equal to $ER_{example}^{TiOSISS}$. So, our proposed scheme not only achieve multiple secret images sharing identical with Liu et al.’s, but also provide the same even more embedding rate than that of theirs.
  (16) $$\begin{array}{cc} ER_{1-bit}^{IHSD-PMSISS} & =\frac{V_{1}\times 256\times 256\times 8+V_{2}\times 128\times 64\times 8}{n\times 256\times 256\times 8}\\ ER_{4-bit}^{IHSD-PMSISS} & =\frac{V_{1}\times 256\times 256\times 8+V_{2}\times 256\times 128\times 8}{n\times 256\times 256\times 8} \end{array}$$
3. **Recovered secret image fidelity.** Our proposed (n,n)-threshold IHSD-MSISS can recover secret images losslessly by applying PSIS or CRTSIS with proper parameters. Both secret images can be recovered without loss in 2-bit payload (3,3)-threshold IHSD-PMSISS. $\left(k_{1},k_{2},n\right)$-threshold TiOSISS scheme can only recover a grayscale secret image losslessly. For the binary secret image, shadow images are stacked to recover, resulting in poor visual quality. Stacking binarized or more shadow images can obtain the recovered secret image with better visual quality. However, this scheme is unable to recover the binary secret image losslessly. Moreover, our proposed scheme realizes a higher embedding rate for the same content as those secret images used in Liu et al.’s, and our recovery operation is much easier.

### 5.4. Discussions

Essentially, our proposed (n,n)-threshold IHSD-MSISS fuses the sharing phases of two secret images in SIS, where we need to consider two sets of ID numbers. The above research focuses on the same sets of ID numbers. In addition, we can adopt different sets of ID numbers, both of which play respective roles in available applications.

**Figure 15:**
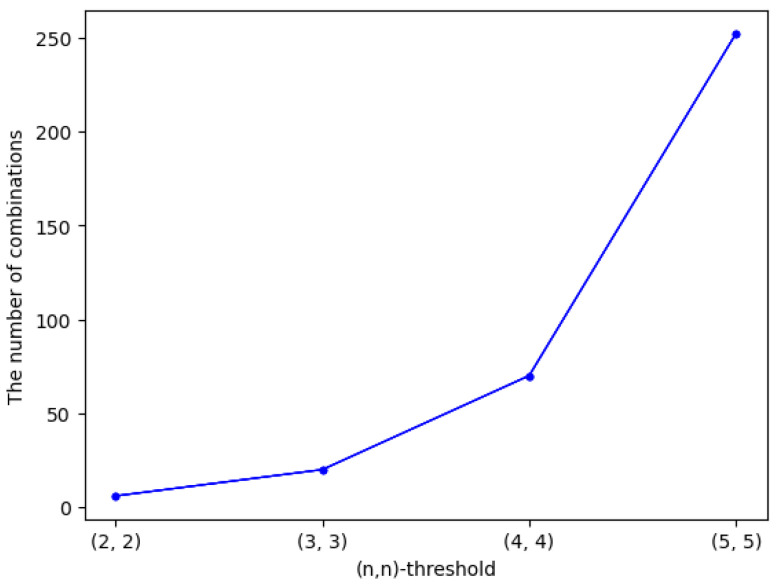
The number of set combinations among different ID numbers.

**Figure 16:**
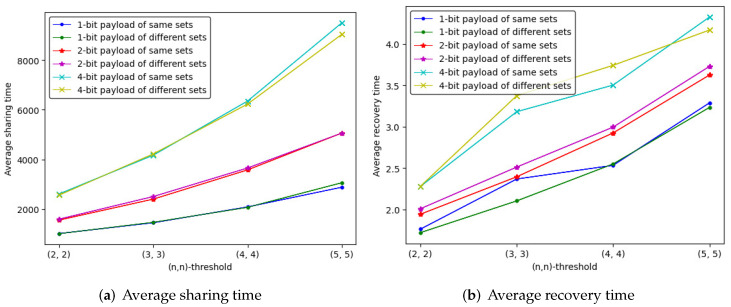
The average time curve of (n,n)-threshold IHSD-PMSISS in three payload situations by same and different ID numbers sets.

1. **Discussion on same ID numbers sets**. Utilizing the same ID numbers sets will save storage, and it is easy to recover secret images. In the sharing phase of our proposed (n,n)-threshold IHSD-MSISS, if we use the same ID numbers set, we only need the storage of one set. Traditionally, sharing two secret images relies on two SISSs, so we have to remember the ID numbers of respective SISS, which will take more storage than ours. Moreover, our scheme will recover two secret images by one set of shares and the corresponding ID numbers set by the same ID numbers. However, with the traditional method, two secret images are reconstructed in the respective independent recovery phase, which is more complex than ours.
2. **Discussion on different ID numbers sets**. Adopting different ID numbers sets, we can improve the security of our proposed (n,n)-threshold IHSD-MSISS. The storage is twice that of using the same ID numbers set. So, it is applicable for the case that we pay more attention to hostile attacks. In (n,n)-threshold IHSD-MSISS, there are 2×n different ID numbers that can be divided into two sets. The possibilities of combination sets are $C_{2\times n}^{n}$, and only one combination can recover two secret images precisely. Through $\left(C_{2\times n}^{n}-1\right)$ sets of ID numbers, we are incapable of obtaining anything about secret images. As *n* is heading up, the more $C_{2\times n}^{n}$ and the harder for attackers. We calculate the number of set combinations and list it in Figure 15.
  The above is discussed in the case that all ID numbers are stored in the same place. However, if we memorize different ID numbers sets in different places, the attackers can misdiagnose using one set of ID numbers. Thus, the difficulty of hostile attacks will be higher. In addition, with different ID numbers sets, we can achieve SIS with weight and SIS with a group, which is applicable by designing the principles that members with different degrees of weight or in different groups will own ID numbers of different sets. Moreover, members with high weight also know the ID numbers managed by those with low weight.
3. **Supplement on ID numbers.** We have analyzed the respective features of ID numbers in two cases. In practical applications, we must consider the differences between using the same ID numbers and different ID numbers. Two parameters, namely, the quantity of available random pixels and average time, are studied.
  - Whether the same ID numbers sets or different ones are used in the proposed (n,n)-threshold IHSD-MSISS, the corresponding quantities of available random pixels are the same, which results from the basic theory of concrete SISS. For example, when we input a PSISS into our proposed scheme and determine the polynomial for a secret pixel, the quantity of available random pixels is also sure, independent of ID numbers.
  - With two cases of ID numbers sets, we experiment with the average sharing and recovery time of (n,n)-threshold IHSD-MSISS in three payload situations, and present the results in Figure 16. From the curves, we can deduce that the average time of utilizing the same ID numbers sets and different ones is similar.

## 6. Conclusions

In this paper, we have proposed a novel and general (n,n)-threshold IHSD-MSISS. The proposed (n,n)-threshold scheme fuses the sharing phases of two secret images by controlling and screening among the randomness of SIS. The introduction of the proposed scheme are presented. We theoretically analyze the practicability and applicability. Through a concrete instance that a (n,n)-threshold IHSD-PMSISS in 1-bit, 2-bit and 4-bit payload, we experiment and further validate the availability and security of the proposed scheme. Comparisons with the scheme of [22] are given for a comprehensive comment on our proposed scheme.

From the experimental and compared results, the proposed scheme can recover two secret images losslessly with PSNR=+∞ and SSIM=1. In the most difficult experimental conditions, (5,5)-threshold with 4-bit payload, the average time is 9497.492 s, which is the longest but still acceptable. In addition, the embedding rate of the proposed scheme in 1-bit payload is equal to that of the scheme [22]. In other, more complex conditions, the embedding rate of the proposed scheme is higher. Thus, the proposed scheme improves the efficiency of sharing multiple secrets.

As the payload bits are increase in number, the current average time by proposed (n,n)-threshold IHSD-MSISS will ascend sharply. Thus, our future work is to improve the efficiency of our proposed scheme. Moreover, we will study IHSD-MSISS on (k,n)-threshold for more applications. 

## Figures and Tables

**Table 1:** The embedding rate.

(n,n)	1-bit	2-bit	4-bit
(2, 2)	0.281	0.312	0.375
(3, 3)	0.188	0.208	0.25
(4, 4)	0.141	0.156	0.188
(5, 5)	0.113	0.125	0.15

**Table 2:** Average sharing time.

(n,n)	1-bit(*s*)	2-bit(*s*)	4-bit(*s*)
(2, 2)	1018.649	1559.642	2611.938
(3, 3)	1454.235	2404.827	4172.467
(4, 4)	2089.951	3579.669	6350.794
(5, 5)	2882.775	5063.865	9497.492

**Table 3:** Average recovery time.

(n,n)	1-bit(*s*)	2-bit(*s*)	4-bit(*s*)
(2, 2)	1.764	1.944	2.279
(3, 3)	2.369	2.396	3.181
(4, 4)	2.534	2.925	3.502
(5, 5)	3.288	3.624	4.323

**Table 4:** Quantity of available random pixels.

(n,n)	1-bit	2-bit	4-bit
(2, 2)	64	16	1
(3, 3)	8256	1032	16
(4, 4)	1,060,912	66,307	259
(5, 5)	136,327,200	4,260,225	4160

## Data Availability

Not applicable.

## Abbreviations

The following abbreviations are used in this manuscript:

SS	Secret sharing
SIS	Secret image sharing
MSIS	Multiple secret images sharing
MSISS	MSIS scheme
IHSD	Information hiding in the sharing domain
IHSD-MSIS	MSIS using IHSD
IHSD-MSISS	IHSD-MSIS scheme
PSIS	Polynomial-based SIS
PSISS	PSIS scheme
IHSD-PMSISS	IHSD-MSISS using PSIS
CRTSIS	Chinese remainder theorem-based SIS
CRTSISS	CRTSTS scheme
VC	Visual cryptography
VCS	VC scheme
RGVCS	Random grid-based VCS
PSNR	Peak Signal-to-Noise Ratio
SSIM	Structural Similarity

## References

[1] Chien M.C., Hwang J.I.G. Secret image sharing using (t, n) threshold scheme with lossless recovery. Proceedings of the 2012 5th International Congress on Image and Signal Processing.

[2] Bao L., Yi S., Zhou Y. (2017). Combination of Sharing Matrix and Image Encryption for Lossless (*k*, *n*) -Secret Image Sharing. IEEE Trans. Image Process..

[3] Thien C.C., Lin J.C. (2002). Secret image sharing. Comput. Graph..

[4] Shyu S.J. (2013). Visual Cryptograms of Random Grids for General Access Structures. IEEE Trans. Circuits Syst. Video Technol..

[5] Yan X., Wang S., Niu X. (2014). Threshold construction from specific cases in visual cryptography without the pixel expansion. Signal Process..

[6] Yan X., Lu Y., Liu L., Wan S., Ding W., Liu H. Chinese Remainder Theorem-Based Secret Image Sharing for (k, n) Threshold. Proceedings of the International Conference on Cloud Computing and Security.

[7] Liu L., Lu Y., Yan X. (2019). Polynomial-based extended secret image sharing scheme with reversible and unexpanded covers. Multimed. Tools Appl..

[8] Yan X., Lu Y., Liu L., Li X., Liu J., Yang G. (2019). Application of Random Elements in Image Secret Sharing. IET Image Process..

[9] Ma Z., Ma Y., Huang X., Zhang M., Liu Y. (2020). Applying cheating identifiable secret sharing scheme in multimedia security. EURASIP J. Image Video Process..

[10] Yan X., Lu Y., Liu L., Song X. (2020). Reversible Image Secret Sharing. IEEE Trans. Inf. Forensics Secur..

[11] Yan X., Lu Y., Liu L. (2020). A Common General Access Structure Construction Approach in Secret Image Sharing. Int. J. Digit. Crime Forensics.

[12] Yan X., Li J., Pan Z., Zhong X., Yang G. (2021). Multiparty verification in image secret sharing. Inf. Sci..

[13] Sun Y., Lu Y., Yan X., Liu L., Li L. (2021). Robust Secret Image Sharing Scheme Against Noise in Shadow Images. IEEE Access.

[14] Iwamoto M., Wang L., Yoneyama K., Kunihiro N., Ohta K. (2006). Visual Secret Sharing Schemes for Multiple Secret Images Allowing the Rotation of Shares. IEICE Trans. Fundam. Electron. Commun. Comput. Sci..

[15] Naidu P.S., Kharat R. Secure Authentication in Online Voting System Using Multiple Image Secret Sharing. Proceedings of the International Symposium on Security in Computing and Communication.

[16] Dastanian R., Shahhoseini H.S. Multi Secret Sharing Scheme for Encrypting Two Secret Images into Two Shares. Proceedings of the 2011 International Conference on Information and Electronics Engineering.

[17] Chen C., Chen J. (2017). A new Boolean-based multiple secret image sharing scheme to share different sized secret images. J. Inf. Secur. Appl..

[18] Sridhar S., Sudha G.F. (2018). Circular meaningful shares based (k, n) two in one image secret sharing scheme for multiple secret images. Multim. Tools Appl..

[19] Prasetyo H., Hsia C. (2019). Improved multiple secret sharing using generalized chaotic image scrambling. Multim. Tools Appl..

[20] Chen T., Wu X. (2020). Multiple secret image sharing with general access structure. Multim. Tools Appl..

[21] Wang J., Yan X., Chen J., Yu Y. (2021). An Intragroup and Intergroup Multiple Secret Images’ Sharing Scheme with Each Participant Holding One Shadow Image. Secur. Commun. Netw..

[22] Liu L., Lu Y., Yan X. (2021). A novel (k1, k2, n)-threshold two-in-one secret image sharing scheme for multiple secrets. J. Vis. Commun. Image Represent..

[23] Chang C.C., Chen Y.H., Chuang L.Y. (2014). Meaningful shadows for image secret sharing with steganography and authentication techniques. J. Inf. Hiding Multimed. Signal Process..

[24] Mao Q., Kb D., Chang C.C. (2016). Novel lossless morphing algorithm for secret sharing via meaningful images. J. Inf. Hiding Multimed. Signal Process..

[25] Xing F., Yan X., Yu L., Sun Y. (2021). Information hiding in the sharing domain (Under revision). J. Vis. Commun. Image Reprresent..

[26] Shamir A. (1979). How to share a secret. Commun. ACM.

[27] Ateniese G., Blundo C., De Santis A., Stinson D.R. (1996). Visual cryptography for general access structures. Inf. Comput..

[28] Liu F., Wu C. (2011). Embedded extended visual cryptography schemes. Inf. Forensics Secur. IEEE Trans..

[29] Yan X., Wang S., Niu X., Yang C.N. (2015). Generalized random grids-based threshold visual cryptography with meaningful shares. Signal Process..

[30] Yang C.N., Wu C.C., Wang D.S. (2014). A discussion on the relationship between probabilistic visual cryptography and random grid. Inf. Sci..

